# 

**Published:** 1892-01

**Authors:** 


### CONTENTS.

ORIGINAL COMMUNICATIONS—

Puerperal Infection—Etiology and Results. By R. R. Kime, M. D., Atlanta, Ga. .......... 641

Electrolysis for the Destruction of Hairs and Moles. By M. B. Hutchins, M. D., Atlanta, Ga. .......... 652

SOCIETY REPORTS—

Southern Surgical and Gynecological Association .......... 656

Gynecological and Obstetrical Society of Baltimore .......... 678

CORRESPONDENCE—

Our New York Letter .......... 682

A Card .......... 687

EDITORIAL—

Dr. L. P. Kenned .......... 689

A Text from a Sermon .......... 689

"Is Drunkenness Curable" .......... 692

Bright's Disease .......... 695

ITEMS .......... 697

SELECTIONS .......... 698

BOOK REVIEWS .......... 704

ITEMS OF INTEREST .......... xi and xii

CLUB RATE PAGE .......... xxxi

INDEX TO ADVERTISEMENTS .......... xiii

